# Physical and nutritional properties of black monkey orange fruit and seeds: A preliminary analysis for food processing

**DOI:** 10.1371/journal.pone.0268628

**Published:** 2022-05-19

**Authors:** Kiana Kirsty van Rayne, Oluwafemi Ayodeji Adebo, Obiro Cuthbert Wokadala, Lucky Sithole, Nomali Ziphorah Ngobese

**Affiliations:** 1 Department of Botany and Plant Biotechnology, University of Johannesburg, Johannesburg, South Africa; 2 Department of Biotechnology and Food Technology, University of Johannesburg, Johannesburg, South Africa; 3 School of Agriculture, University of Mpumalanga, Nelspruit, South Africa; 4 Department of Agriculture and Rural Development, Pietermaritzburg, South Africa; Bangabandhu Sheikh Mujibur Rahman Agricultural University, BANGLADESH

## Abstract

*Strychnos madagascariensis* is an underutilized South African fruit-bearing tree, with the pulp being the primary consumable component. However, the seeds hold the potential as a food source due to their high nutrient composition. The aim of this study was to determine the physical properties of *S*. *madagascariensis* fruit and seeds to aid in food processing equipment development. Fruit physical properties were determined at four progressive ripening stages, as well as the seed physical properties and mineral composition. The pulp contributed the most towards fruit composition across stages of ripeness (*c* 50%), followed by the rind (*c* 30%) and seeds (*c* 20%). Furthermore, significant variations in seed physical properties were observed at progressive maturity stages. The seeds showed significantly greater mineral compositions in unripe-green fruit in comparison to fruit at progressive ripening stages. The data provided may serve as a basis for the development of processing procedures and equipment and suggests that seeds of unripe-green fruit hold greater nutritional benefits.

## Introduction

Indigenous wild food plants have proven to be a resilient source of nutrition, especially in times of food scarcity (due to natural or man-made disasters). However, these food sources have been stigmatized as ‘famine food’ and thus neglected as a primary source of nutrition. The underutilization of these indigenous species may also be due to the lack of information surrounding their nutritional value and potential in mitigating food insecurity [1]. Moreover, the consumption of wild indigenous food plants may aid in the alleviation of mineral deficiencies, which is prevalent in Africa.

Minerals are essential elements that are required for the proper functioning of the human body. These minerals are subdivided into major or macro-minerals and trace or micro-minerals based on the quantities required to support healthy diets. The major minerals encompass elements such as calcium (Ca), magnesium (Mg), potassium (K), sodium (Na), chloride (Cl), phosphorus (P) and sulphur (S); while the subcategory of trace minerals includes elements such as iodine (I), zinc (Zn), selenium (Se), iron (Fe), manganese (Mn), copper (Cu), cobalt (Co), molybdenum (Mo), fluoride (F), chromium (Cr) and boron (B) [2]. Apart from combating diseases particularly associated with mineral deficiencies, certain minerals (Ca, Mg, Cu, Se and Zn) are of importance for optimal functioning of the immune system. Thus, they may aid in mitigating the effects of pandemics such as SARS-CoV-2 and promoting faster recovery [3].

The native southern African fruit-bearing tree, *Strychnos madagascariensis* (more commonly known as black monkey orange) is highly valued in the rural communities surrounding the region in which this species grows [4]. The fruit pulp is typically consumed raw as a snack or is processed into other value-added food products (such as fruit rolls, powders, jams, or juices) [5, 6]. This crop is valued based on its high fruit yields, fruiting seasons, drought tolerance and wide distribution across the northern and eastern regions of southern Africa [7]. However, despite the importance of the black monkey orange fruit in rural households (especially during times of food scarcity), this fruit is considered to be an underutilized food commodity [7]. This is partially due to consumer preferences, as other species within this genus (*S*. *cocculoides*, *S*. *spinosa*, and *S*. *pungens*) are selected based on the taste of the fruit pulp [8]. Although the seeds of *S*. *madagascariensis* are avoided due to the presence of the toxic alkaloid strychnine, they hold the potential as a food source due to their high sugar, fiber, potassium and iron contents [7, 9].

The utilization of seeds as food alternatives or supplements have recently gained popularity to reduce food wastage and improve dietary nutrition [10]. It has been noted that fruit processing companies generate large quantities of seeds as waste products and thus, opening the market for the development of seed-based food ingredients [10]. Currently, food industries are using waste seeds as a means of boosting the nutritional value and improving the sensory quality of their food products, as well as the development of new food products. Additionally, the utilization of waste seed may reduce the cost of waste management and processing due to the overall decrease in seed waste production. It may also be beneficial to the environment since large quantities of waste from food processing is improperly discarded [11, 12]. Some examples of seeds used as food supplements include papaya seed flour, used to improve the nutritional and sensory quality of hamburger meat [11], and pumpkin seed flour, used in improving the nutritional quality of baked goods [12].

The physical properties of seeds are important parameters in the development of processing procedures (such as harvesting, transport, storage, cleaning, drying, milling and freezing) as well as in the development of machinery to make these processes more streamlined. The development of machinery requires knowledge of seed shape, size surface area and other characteristics to process material from harvest to table [13, 14]. Thus, the aim of this research is to provide the basic physical properties of *S*. *madagascariensis* seeds to serve as a reference point for processing procedures and equipment development as well as assessing the changes that may occur in the mineral and nutrient composition of the seeds during ripening.

## Materials and methods

### Sampling

Fruit of *S*. *madagascariensis* were collected at four stages of maturity (unripe, semi-ripe, ripe, and overripe) from Bushbuckridge, South Africa (24°59ʹ26.3˝ S, 31°09ʹ36.9˝ E). The second batch of fruit at two stages of ripeness (unripe and ripe) were collected from Shongweni Nature Reserve, South Africa (29°85.5ʹ9780˝ S, 30°72.9ʹ4250˝ E). A total of 50 monkey orange fruits were collected from five different trees (per maturity stage), in the same location. Permission to collect plant samples was granted by the South African Department of Environment, Forestry and Fisheries (DEFF)—BABS/001020N.

### Fruit physical properties

The fruit weights were measured using a digital scale (Kern, Germany), the size (circumference) was measured using a measuring tape and a CR-10 Plus chromameter (Konica Minolta, Japan) was used to measure the colour at three random regions of the fruit rind at each stage of maturity. The seeds were then removed from the fruit and separated from the pulp, the weight of each fruit component (rind, pulp and seeds) was measured to determine the composition of the fruit (%rind, %pulp and %seed).

### Seed physical properties

#### Axial dimensions

**Fig 1:**
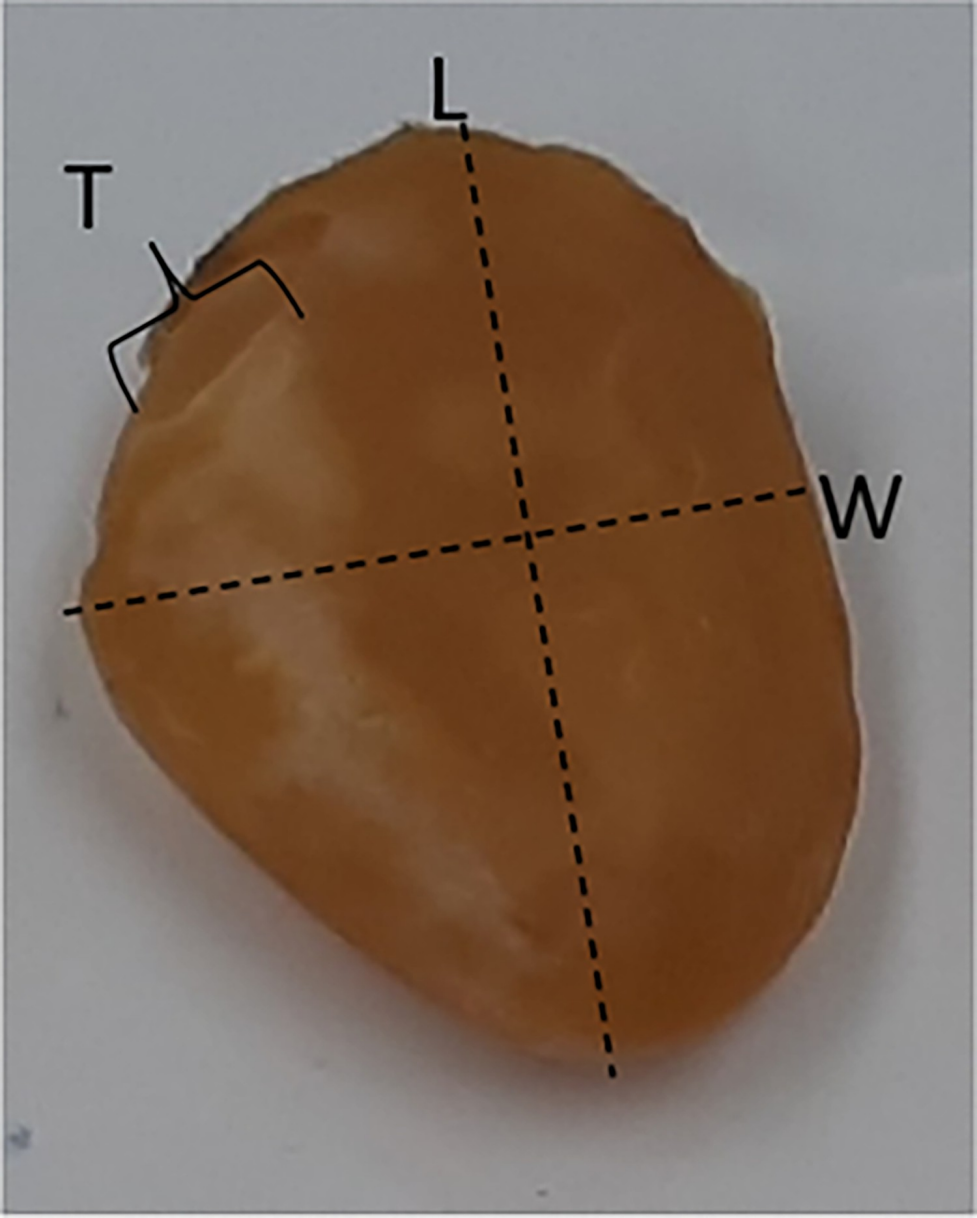
A representation of the axial dimensions of *S*. *madagascariensis* seeds.

The average seed size was determined by randomly selecting 60 seeds. The length (L), width (W) and thickness (T) of each seed were measured using a dial vernier calliper. The length was measured as the longest portion of the seeds while the perpendicular measurement was deemed as the width (Fig 1). Lastly, the thickness of the seeds was identified as the smallest feature of the three dimensions. Once these measurements were taken, the seeds were stored in a freezer (at -20°C).

#### Sphericity

This was determined using the practical 3-dimensional expression, whereby the higher sphericity values indicate more spherical samples. This is particularly important in the development of hopper and dehulling equipment [21]. The sphericity of *S*. *madagascariensis* seeds was calculated using Eq 1. Where Ø represents sphericity and other parameters defined above.


$$\emptyset =\frac{{\left(LWT\right)}^{\frac{1}{3}}}{L}$$ (1)

#### Aspect ratio

This parameter represents the relationship between width (W) and length (L) and was calculated using Eq 2.


$$Aspect\;ratio=\frac{W}{L}$$ (2)

#### Geometric mean diameter

This parameter was calculated using the axial dimensions of the seeds and Eq 3. Where Gm represents the geometric mean.


$$\mathrm{Gm}={\left(\mathrm{LWT}\right)}^{\frac{1}{3}}$$ (3)

#### Surface area

An estimate of the surface area of the seeds was calculated using Eq 4. Where S represents the surface area (mm^2^) and Gm the geometric mean diameter (mm)

$$S={\pi Gm}^{2}$$ (4)

#### Roundness

The roundness [30] of the seeds were calculated using the formula below, where W represents the width, L represents length and T represents thickness.


$$Roundness=\frac{\left[(\frac{W}{L})+(\frac{T}{L})+(\frac{T}{W})\right]}{3}$$ (5)

### Seed nutritional properties

#### Mineral analysis

The micronutrient content of the seeds of *S*. *madagascariensis* was determined using Inductively Coupled Plasma–Optical Emission Spectrometry (ICP-OES). Samples (0.25 g, dry weight) were ashed in a furnace (ULTRA–FURN, Johannesburg) at 450°C for 24 h. Once the samples were cooled, they were digested using 2 mL of 32% HCL (Associated Chemical Enterprises, Johannesburg). The samples were then evaporated to dryness using a water bath, set at 90°C, model 402 (Scientific, South Africa). Once the samples were dry, 25 mL of freshly prepared 1:9 diluted HCL was added to each sample and the mixture was then filtered using an Advantec 5B:90 mm diameter filter paper. The filtrate was then diluted using de-ionized water (5:20) and analysed using the VISTA-MPX CCD simultaneous ICP-OES (SMM Instruments, South Africa).

The proximate analysis of *S*. *madagascariensis* Location 1 seeds was done as follows: protein was determined by multiplying the total nitrogen content by the conversion factor 6.25, ether extraction was used for fats, and fiber was determined through acid/neutral detergent analysis [7]. The total carbohydrate content was calculated through difference while the total energy component was calculated using the protein, fat and carbohydrate contents. The method of analysis used for the quantification of the mineral components was atomic absorption spectrophotometry (AAS). Seeds from Location 2 were not analyzed due to limited resources.

### Statistical analysis

IBM SPSS Statistics Data Editor 27.0 was used for statistical analysis. A one-way repeated measures ANOVA was performed to differentiate between means at a 95% probability level. Descriptive statistics were also obtained from the IBM SPSS software. A minimum of four replicates were used for mineral analysis, 60 replicates were used for the seed physical properties and 50 replicates were used for the fruit physical properties.

## Results

### Fruit physical properties

**Fig 2:**
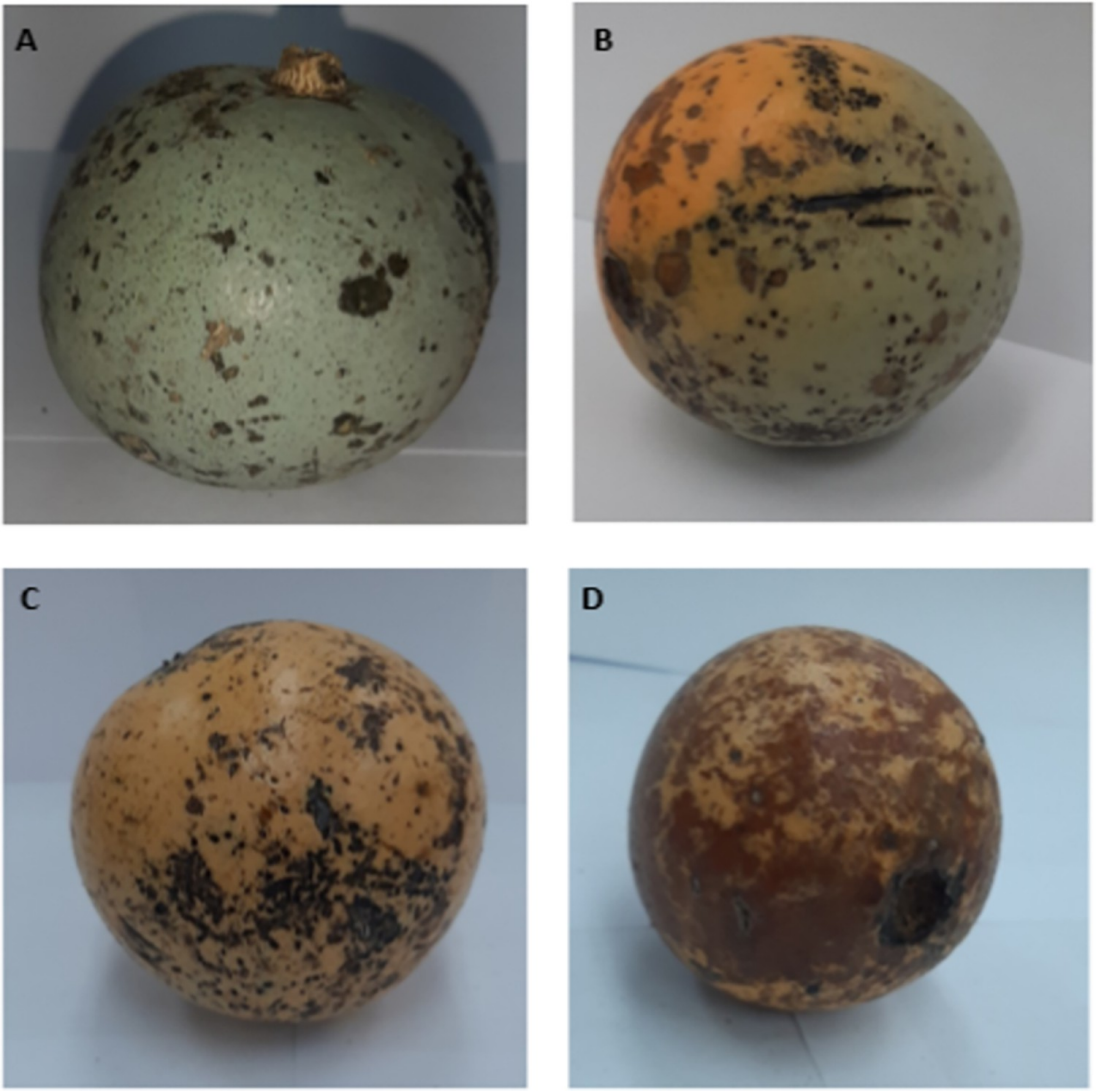
*Strychnos madagascariensis* fruit at four stages of ripeness. (A) stage 1 –unripe-green fruit; (B) stage 2 –semi-ripe fruit; (C) stage 3 –ripe fruit; (D) stage 4 –over ripe fruit.

The physical properties of *S*. *madagascariensis* fruit, at four stages of ripeness, are documented in Table 1 and Fig 2. The fruit showed a significant (P < 0.05) decrease in weight from stages 1 and 2, with a non-significant (P > 0.05) increase in weight at stages 3 and 4. A similar trend was observed in fruit diameter, where a significant decrease in fruit diameter occurred between stages 1 and 2, with a non-significant increase occurring at stage 4. The fruit colour was measured using the CIE LAB scale, where parameters such as the L* value, a* value and b* value was measured. The degree of lightness (L* value) of the fruit rind showed a significant increase between stages 1, 3 and 4, with a non-significant increase occurring at stage 2. The fruit rind showed a colour shift from green to red between stages 2 and 3, with significant differences occurring between stages 1, 3 and 4). Lastly, the degree of yellowness (b* value) showed a significant increase from stage 1 to stage 4. The fruit composition of *S*. *madagascariensis* was evaluated at four stages of ripeness and was determined based on the percentage contribution towards the total fruit weight. The pulp composition remained relatively constant throughout all four stages (Table 1), except for a significant (P < 0.05) increase occurring between stage 1 and 3. The fruit rind showed a general decline in percentage composition as the fruits progressed from stage 1 to stage 4, with significant decreases occurring at stages 3 and 4. Lastly, the percentage composition of the seeds displayed a general increase with the progression of ripeness, where statistically significant increases were observed between stages 1 and 4.

**Table 1:** Physical properties of *S*. *madagascariensis* fruit. The means for each parameter are reported under the respective stages of maturity followed by the standard error in brackets. Means accompanied by the same letter in a given column are not significantly different (P > 0.05).

Stage	Weight (g)	Diameter (cm)	Colour			Composition (%)		
			L*	a*	b*	Pulp	Rind	Seed
1	286.96 (23.23) ^a^	8.07 (0.21) ^a^	56.98 (0.27) ^a^	-5.65 (0.13) ^a^	18.83 (0.34) ^b^	49.48 (0.92) ^a^	33.32 (1.16) ^a^	17.20 (0.96) ^ab^
2	160.20 (7.89) ^c^	6.48 (0.11) ^c^	57.65 (0.21) ^a^	-5.84 (0.09) ^a^	15.86 (0.30) ^c^	51.96 (1.83) ^ab^	30.01 (1.27) ^ab^	15.02 (0.71) ^a^
3	230.76 (13.52) ^ab^	7.52 (0.16) ^ab^	69.68 (0.53) ^b^	18.48 (0.25) ^b^	46.71 (0.25) ^a^	53.13 (0.44) ^b^	29.30 (0.53) ^bc^	17.57 (0.28) ^b^
4	201.60 (10.81) ^b^	7.12 (0.13) ^b^	66.47 (0.26) ^c^	18.24 (0.14) ^b^	45.90 (0.35) ^a^	49.11 (0.90) ^a^	29.08 (0.92) ^c^	21.81 (0.69) ^c^

Colour changes in the fruit rind at progressive maturity stages are visually observed in Fig 2, where the fruit rind colour changed from green to orange/yellow (Fig 2B and 2C). These colour changes were numerically measured and documented in Table 1. Image A represents completely unripe-green fruits (stage 1) where the rind is completely blue green. Image B represents fruits that have begun to ripen (stage 2) where the rind is partially yellow/orange. Image C represents fruit that are completely ripe (stage 3) with an orange rind and image D represents fruit that are over-ripe (stage 4) with an orange/brown rind.

### Seed physical properties

The axial dimensions (length, width, and thickness) of *S*. *madagascariensis* seeds at four stages of ripeness are presented in Table 2. The seeds showed a significant (P < 0.05) decrease in length from fruits collected at stage 1 to stage 4, with a non-significant increase in length at stage 2. The seeds also showed a significant increase in width between stages 1 and 4, while a non-significant increase and decrease occurred at stages 2 and 3, respectively. Lastly, the seeds showed significant increases in thickness, from stages 1 to 4. The parameters for seed shape (sphericity, aspect ratio, geometric mean diameter, surface area and roundness) are presented in Table 2. The sphericity of the seeds showed significant increases from stage 1 to stage 4. The aspect ratio of the seeds showed a significant increase between stages 1 and 4 only, with non-significant increases occurring at stages 2 and 3. The geometric mean diameter showed significant increases between stages 1, 2 and 4, with a non-significant increase occurring at stage 3. The surface area of the seeds followed a similar trend to that observed in the geometric mean diameter. Lastly, the degree of roundness of the seeds showed significant increases between stages 1, 3 and 4, with a non-significant increase occurring at stage 2.

**Table 2:** Axial dimensional properties and shape of *S*. *madagascariensis* seeds at four stages of ripeness. The means for each parameter are reported under the respective stages of maturity followed by the standard error in brackets. Means accompanied by the same letter in a given row are not significantly different (P > 0.05).

Parameters	Stage 1	Stage 2	Stage 3	Stage 4
**Length (cm)**	2.48 (0.01) ^a^	2.50 (0.01) ^a^	2.41 (0.01) ^b^	2.41 (0.01) ^b^
**Width (cm**	1.91 (0.01) ^ab^	1.95 (0.01) ^ac^	1.89 (0.01) ^b^	1.99 (0.02) ^c^
**Thickness (cm)**	0.88 (0.01) ^b^	0.92 (0.01) ^a^	0.93 (0.01) ^a^	1.00 (0.01) ^c^
**Sphericity**	0.65 (0.00) ^a^	0.66 (0.00) ^b^	0.67 (0.00) ^c^	0.70 (0.00) ^d^
**Aspect ratio**	0.77 (0.01) ^a^	0.78 (0.01) ^a^	0.79 (0.01) ^a^	0.78 (0.01) ^b^
**Geometric mean diameter (mm)**	16.00 (0.07) ^a^	16.46 (0.07) ^b^	16.16 (0.07) ^a^	16.80 (0.07) ^c^
**Surface area (mm** ^**2**^ **)**	809.17 (7.27) ^a^	855.91 (7.16) ^b^	825.02 (6.92) ^a^	890.36 (7.08)^c^
**Roundness**	0.53 (0.00) ^a^	0.55 (0.00) ^a^	0.56 (0.00) ^b^	0.59 (0.00) ^c^

### Seed nutritional properties

Table 3 shows a comparison in mineral content of the seeds of *S*. *madagascariensis* at four stages of fruit ripeness (i.e. unripe-green fruit, partially ripe–green/yellow fruit, ripe–orange fruit and overripe–orange/brown fruit). Minerals such as nitrogen, potassium, manganese, and iron, contribute the most in terms of mineral composition across all four stages of ripeness. The seeds show a decreasing trend in mineral composition as the fruits progress from stage 1 to stage 4, with significant differences (P < 0.05) occurring between these stages for nitrogen, calcium, magnesium, potassium, sodium, manganese, and iron. No significant differences occurred between stages 2 and 3 for all minerals. Table 4 shows a comparison in mineral composition between the seeds of the fruit that were collected from two localities (i.e. location 1—Bushbuckridge and location 2—Shongweni Nature Reserve) and two stages of ripeness (stage 1 –unripe -green and stage 2 –ripe). Seeds collected from the second location for both stages of ripeness show a significantly lower mineral composition, except in zinc, copper, phosphorous and aluminium. Moreover, a significant increase between location 1 and 2, in the iron content, at stage 1 was observed, as well as in the magnesium and potassium contents at stage 3.

**Table 3:** Mineral composition of *S*. *madagascariensis* seeds at four stages of ripeness. The means for each parameter are reported under the respective stages of maturity followed by the standard error in brackets. Means accompanied by the same letter in a given column are not significantly different (P > 0.05). This information is based on seeds collected from location 1: Bushbuckridge.

Stage	Minerals (%)										
	N	Ca	Mg	K	Na	Zn	Cu	Mn	Fe	P	Al
1	1.65(0.20) ^a^	0.11(0.0) ^a^	0.11(0.0) ^c^	0.90(0.1) ^b^	0.03(0.00) ^a^	0.000(0.0006) ^a^	0.0006(0.0001) ^ab^	0.011(0.001) ^b^	0.0016(0.0011) ^a^	0	0.0001(0.0002) ^a^
2	1.38(0.2) ^b^	0.10(0.0)^a^	0.09(0.0) ^ab^	0.72(0.1) ^a^	0.02(0.01) ^ab^	0.0006(0.0006) ^a^	0.0005(0.0001) ^ab^	0.008(0.001) ^a^	0.0009(0.0006) ^a^	0	0.0002(0.0004) ^a^
3	1.41(0.18) ^ab^	0.11(0.0)^a^	0.09(0.0) ^a^	0.58(0.0) ^a^	0.01(0.00) ^b^	0.0010(0.0003) ^a^	0.0007(0.0001) ^a^	0.008(0.001) ^a^	0.0045(0.0066) ^ab^	0	0.0001(0.0002) ^a^
4	1.32(0.0) ^b^	0.07(0.0) ^b^	0.10(0.0) ^b^	0.54(0.0) ^c^	0.00(0.00) ^c^	0.0009(0.0001) ^a^	0.0004(0.0001) ^b^	0.005(0.00) ^c^	0.0041(0.0007) ^b^	0	0.0001(0.0001) ^a^

**Table 4:** Comparison of the mineral composition of *S*. *madagascariensis* seeds collected from different locations at two stages of ripeness. The means for each parameter are reported under the respective stages of maturity followed by the standard error. Means accompanied by the same letter in a given column are not significantly different (P > 0.05). Location 1: Bushbuckridge, Location 2: Shongweni Nature Reserve.

Stage	Location	Minerals (%)										
		N	Ca	Mg	K	Na	Zn	Cu	Mn	Fe	P	Al
1	1	1.65(0.20) ^a^	0.11(0.01) ^a^	0.11(0.01) ^a^	0.90(0.13) ^a^	0.03(0.007) ^a^	0.001(0.0001) ^a^	0.001(0) ^a^	0.011(0.001) ^a^	0.002(0.001) ^a^	0	0.0001(0.0002) ^a^
	2	1.13(0.13) ^b^	0.05(0.01) ^b^	0.10(0.01) ^b^	0.66(0.09) ^b^	0.01(0.001) ^b^	0.001(0.0001) ^a^	0.001(0) ^a^	0.006(0.000) ^b^	0.005(0.002) ^b^	0	0.0005(0.0006) ^a^
3	1	1.41(0.18) ^a^	0.12(0.01) ^a^	0.09(0.00) ^a^	0.58(0.02) ^a^	0.01(0.003) ^a^	0.001(0.0003) ^a^	0.001(0) ^a^	0.008(0.001) ^a^	0.005(0.007) ^a^	0	0.0001(0.0002) ^a^
	2	1.22(0.16) ^b^	0.05(0.01) ^b^	0.10(0.00) ^b^	0.69(0.06) ^b^	0.01(0.001) ^b^	0.001(0.0001) ^a^	0.001(0) ^a^	0.005(0.001) ^b^	0.005(0.002) ^a^	0	0.0001(0.0002) ^a^

## Discussion

The physical properties of fruits make up some of the most essential parameters in the design of postharvest processes such as fruit grading, transportation as well as packaging. Physical properties of produce also play a role in engineering applications, as it forms part of a collection of parameters valuable for engineering purposes (including mechanical, electrical, thermal, light, acoustic and chemical properties). Physical parameters such as dimensions and weight are typically used in sizing systems [15]. The fruit physical properties of *Strychnos madagascariensis* are shown in Table 1. The average fruit weights from stages 1 to 4 are approximately 286.96 g, 160.20 g, 230.76 g and 201.60 g, respectively. Significant differences (P < 0.05) were observed between stages 1 and 2, stages 1 and 4, stages 2 and 3, stages 2 and 4 and stages 3 and 4. These variations may be a result of the area in which the sample trees are grown or the size and age of the trees. These weights show similarities to that of citrus fruit such as oranges (var. Tompson), which have a weight range of 159.76 g– 277.53 g (small to large-grade oranges) [15]. Similar variations occurred in fruit diameter from stages 1 to 4, fruits showing 8.07, 6.48, 7.52 and 7.12 cm, respectively. Significant differences were observed between the same stages as the fruit weight. Again, the fruit diameters of *S*. *madagascariensis* show similarities to that of oranges, which range from 7.79 cm to 9.04 cm [15]. Based on the similarities shared with oranges, similar postharvest processes such as transportation from the site of harvest to processing companies and packaging containers may be adopted for *S*. *madagascariensis* fruits. When looking at the fruit composition, the fruit pulp contributed the most in terms of percentage weight, followed by the fruit rind and seeds. This remained a consistent trend, irrespective of the stage of ripeness of the fruit. The pulp, from stage 1 to 4, makes up approximately 49.48%, 51.96%, 53.13% and 49.11%, respectively with significant differences only occurring between stages 1 and 3 and stages 3 and 4. The decrease in pulp weight at stage 4 of ripeness may be due to water loss as the fruit begins to senesce. Furthermore, the fruit rind makes up 33.32%, 30.01%, 29.30% and 29.08%, respectively. Lastly, the seeds from stage 1 to 4, contributes approximately 17.20%, 15.02%, 17.57% and 21.81% respectively; with significant differences mainly occurring at stage 4, for both the fruit rind and seeds.

Colour and taste play an important role in the acceptability of foods [16]. These qualities are often a result of phytochemical pigments with antioxidant activity [17, 18]. When assessing the colour changes (Table 1 and Fig 2) of the fruit rind at progressive ripening stages, it was observed that the L* value, representing the degree of lightness, increased from stage 1 to 4; with significant differences occurring between stages 1, 3 and 4. The fruits also showed a colour shift from green to red, indicated by the a* value, with significant differences occurring between stages 1, 3 and 4. This colour change is visually observed in Fig 2B and 2C, where the fruit rind becomes completely orange/yellow. Lastly, the fruits showed an increase in the degree of yellowness, as indicated by the b* value, with significant differences occurring from stages 1 to 4. Colour changes during the progression of ripeness of the fruit may be a valuable tool in the development of maturity indices for identifying the most optimal stage of harvesting and consumption. This was shown to be the case for a late season Spanish peach cultivar Calanda by Ferrer *et al*. [19], who reported the increase in yellowness to be associated with an increase in carotenoids, carotene and xanthophylls during maturation. The development of yellowness has been shown to coincide with the degradation of chlorophylls in fruits with a similar colour development pattern like lemons, which turn from green to yellow during ripening [20].

Axial dimensions (length, width, and thickness) of *Strychnos madagascariensis* seeds at four stages of ripeness are shown in Table 2. The length of the seeds from stages 1 to 4 ranged from 1.91 cm to 3.22 cm, 0.94 cm to 3.19 cm, 1.78 cm to 3.10 cm and 1.90 cm to 3.00 cm, respectfully. While significant differences (P < 0.05) were observed between stages 1 and 3, stage 1 and 4, stage 2 and 3 as well as stage 2 and 4, the length of seeds from stages 1 and 2 and those of stages 3 and 4 were similar. The width of the seeds from stages 1 to 4 ranged from 1.19 cm to 2.98 cm, 1.21 cm to 2.98 cm, 0.95 cm to 2.26 cm and 1.46 cm to 2.50 cm, respectively. Significant differences were observed between stage 1 and 4, stage 2 and 3 as well as stage 3 and 4. Lastly, the thickness of the seeds from stages 1 to 4 ranged from 0.60 cm to 1.91 cm, 0.60 cm to 1.20 cm, 0.57 cm to 1.29 cm and 0.64 cm to 1.81 cm. Significant differences were observed between all stages of ripeness, except between stages 2 and 3. From the statistical analysis, it can be seen that significant differences in the axial dimensions of the seeds mostly occurs between stages 1 and 4. Axial dimensions act as a useful tool in determining the volume of the seeds, which is a central input for designing seed cracking and dehulling equipment as demonstrated for locust bean seed [21].

The dimensional properties (sphericity, aspect ratio, geometric mean diameter, surface area and roundness) of *Strychnos madagascariensis* seeds at four stages of ripeness are shown in Table 2. The average sphericity of the seeds from stages 1 to 4 are 0.65, 0.66, 0.67 and 0.70, respectively. The seeds of *S*. *madagascariensis* show similarities in sphericity to that to that of almond nuts, 62.96% (0.6296) [22]. Statistical analysis showed no significant differences (P > 0.05) between the four stages of ripeness. The average aspect ratio of the seeds from stages 1 to 4 is 0.77, 0.78, 0.79 and 0.78, respectively. Significant differences (P < 0.05) were observed between stage 1 and 4, stage 2 and 4 as well as stage 3 and 4. The geometric mean diameter of the seeds from stages 1 to 4 were 16.00, 16.46, 16.16 and 16.80 mm. Which shows similarities to that of fresh litchi seeds, 16.80 cm [23]. Significant differences were observed among all stages, except between stages 1 and 3. The average surface area of the seeds from stages 1 to 4 was 809.17, 855.91, 811.84 and 825.64 mm^2^. Similar to the geometric mean diameter, significant differences were observed between all stages of ripeness, except between stages 1 and 3. Lastly, the average roundness of the seeds from stages 1 to 4 was 0.53, 0.54, 0.56 and 0.59. Significant differences only started occurring from stage 2 onwards, with no significant differences occurring between stages 1 and 2.

As previously mentioned, *S*. *madagascariensis* is primarily targeted for its pulp for consumption however, one study has shown that the seeds have the potential for human nutrition. The seeds of *S*. *madagascariensis* have shown the potential as a possible food source due to its high fiber, sugar, iron, and manganese content [7]. The current study shows that the mineral composition of *S*. *madagascariensis* seeds varies between stages of ripeness (Table 3). The nitrogen, potassium, manganese and iron content of the seeds was found to be the highest at the unripe-green stage (stage 1): quantified to be 1.6466; 0.9017; 0.0107; and 0.0016 g/ 100 g, respectively with significant decreases occurring between stages 1 and 2 and stages 1 and 4. The potassium and iron contents found in the seeds of *S*. *madagascariensis* were substantially higher than that found within whole-grain wheat flour (0.3630 and 0.0041, respectively) [24]. The calcium content was found to be the highest at stage 1 (0.1085 g/100 g) with a significant decrease in composition occurring between stages 1 and 4 only. The magnesium content was found to be the highest at stage 1 (0.1032 g/100 g), with a significant decrease occurring between stages 1 and 2 only. The sodium content of the seeds was the highest at stage 1 of ripeness (0.0213 g/100 g), weight significant decreases occurring between stages 1 and 3 and stages 1 and 4. The manganese content was the highest at stage 1 of ripeness (0.0105 g/100 g), with significant decreases occurring with progressive ripening of the fruit.

Table 5 Compares the macronutrient composition of the seeds at progressive ripening stages. The major components contributing towards the macronutrient composition, across all four stages of ripeness of the seeds, are fiber (77.59% - 80.02%) and protein (8.28% - 10.29%). However, van Rayne *et al*. [7], reported that the major components are fiber (53%), sugar (41%) and protein (8.27%). As can be seen in Table 5, parameters such as total energy, moisture content, total fat and total fiber, showed no significant changes at progressive ripening stages. However, the ash content showed significant decreases (primarily observed at stages 3 and 4). This correlates with the decreasing trend observed in mineral composition (Table 3). Significant decreases were also observed in the seed’s protein content at progressive ripening stages. Moreover, significant increases in the total carbohydrate fraction were observed. This may be due to the accumulation of sugars within the seeds as an increase in sweetness at progressive ripening stages is a common characteristic in the ripening of climacteric fruits [25]. Alternatively, increases in sugar content may also be due to the plant’s response to drought stress. As drought-tolerant plants react to drought stress through the accumulation of osmoprotectant compounds, such as proline, glycine betaine, polyamines and sugars [26]. This may also explain the high percentage of sugars reported by van Rayne *et al*. [7], as the sampling trees used in that study may have been experiencing severe drought stress, resulting in an increase in sugars.

**Table 5:** Proximate composition of *S*. *madagascariensis* seed flour (per 100 g, dry mass basis) at four stages of ripeness. Data presented in the table above represents the mean of five replicates, followed by the standard error. Values associated with the same letter in each row for each location show no significant differences (P < 0.05).

Composition	Stage 1	Stage 2	Stage 3	Stage 4
**Total Energy (Kcal)**	73.24(12.25) ^a^	84.23(21.52) ^a^	84.47(8.78) ^a^	87.14(12.10) ^a^
**Moisture (%)**	7.39(0.30) ^a^	7.48(0.35) ^a^	7.48(0.30) ^a^	6.55(0.39) ^a^
**Protein (%)**	10.29(1.25) ^a^	8.63(1.22) ^b^	8.78(1.14) ^ab^	8.28(0.17) ^b^
**Total fat (%)**	0.61(0.20) ^a^	0.55(0.25) ^a^	0.54(0.16) ^a^	0.58(0.18) ^a^
**Total carbohydrates (%)**	86.97(1.15) ^b^	89.02(1.07) ^ab^	88.75(1.24) ^ab^	90.03(0.45) ^a^
**Total fiber (%)**	80.02(3.12) ^a^	77.59(5.48) ^a^	77.71(2.29) ^a^	77.92(2.98) ^a^
**Ash (%)**	2.43(0.26) ^a^	2.04(0.25) ^ab^	1.85(0.02) ^b^	1.02(0.29) ^c^

## Conclusion

This research was conducted to expand the knowledge surrounding the fruits and seeds of *S*. *madagascariensis*, provide basic physical parameters for food processing and assess changes in mineral composition across stages of ripeness. The data presented in this research may serve as a basis for the development of harvesting and processing procedures and equipment. The fruit physical properties (weight, diameter, pulp, and rind composition) showed a general decrease between stages 1 and 4. While the seed parameters (seed composition, width, thickness, sphericity, aspect ratio, geometric mean diameter, surface area, roundness) showed a general increase between stages 1 and 4. Thus the development of processing procedures and equipment may be dependent on the stage of ripeness targeted for food processing. Furthermore, the seeds showed higher mineral, protein, fat and fiber compositions at stage 1 of ripeness (unripe-green fruit) suggesting that processing of the seeds at this stage may provide a higher nutritional benefit. Further research is required surrounding the detoxification processes of the seeds to improve their suitability for human consumption. In addition, research in the identification of rind pigments based on colour and the possible benefits of these pigments should be conducted. Studies on shelf-life should be conducted to possibly extend the shelf-life of the fruit after harvest (as it was observed in this study that the fruits ripened quickly).

## Supporting information

S1 Data (XLSX)
